# Dihydromyricetin-Encapsulated Liposomes Inhibit Exhaustive Exercise-Induced Liver Inflammation by Orchestrating M1/M2 Macrophage Polarization

**DOI:** 10.3389/fphar.2022.887263

**Published:** 2022-06-02

**Authors:** Xi Zhou, Long Yi, Hedong Lang, Jun Zhang, Qianyong Zhang, Li Yu, Jundong Zhu, Mantian Mi

**Affiliations:** Research Center for Nutrition and Food Safety, Chongqing Key Laboratory of Nutrition and Food Safety, Institute of Military Preventive Medicine, Third Military Medical University (Army Medical University), Chongqing, China

**Keywords:** dihydromyricetin, exhaustive exercise, liposome, liver inflammation, macrophage polarization

## Abstract

Exhaustive exercise (EE) induced hepatic inflammatory injury has been well reported. Dihydromyricetin (DHM) has shown anti-inflammatory bioactivity and hepatoprotective effects but is limited by poor bioavailability. Here, high-bioavailability DHM-encapsulated liposomes were synthesized and explored for their therapeutic potential and regulatory mechanisms in a hepatic inflammatory injury model. The animal model was established by swimming-to-exhaustive exercise in C57BL/6 mice, and the anti-inflammatory effects were detected after administration of DHM or DHM liposome. NIR fluorescence imaging was used to assess the potential of liver targeting. The DHM liposome-induced macrophage polarization was measured by flow cytometry *ex vivo*. The anti-inflammatory mechanism of DHM was studied in cell line RAW264.7 *in vitro*. Liposome encapsulation enhanced DHM bioavailability, and DHM liposome could alleviate liver inflammation more effectively. Moreover, DHM liposome targeted hepatic macrophages and polarized macrophages into an anti-inflammatory phenotype. The SIRT3/HIF-1α signaling pathway could be the major mechanism of DHM motivated macrophage polarization. Our study indicates that DHM liposomes can alleviate liver inflammation induced by EE through sustained releasing and hepatic targeting. It is a promising option to achieve the high bioavailability of DHM. Also, this study provides new insights into the regional immune effect of DHM against inflammation.

## 1 Introduction

Many studies have reported that the appropriate frequency and intensity of exercise benefit public physical quality. Suitable exercise exerts a favorable effect on many metabolic disorders like diabetes mellitus type 2 and angiocardiopathy (Fan and Evans, 2017). Nevertheless, exhaustive exercise (EE) of extensive intensity and duration, e.g., marathon and long-distance swimming, might do harm to fitness. Such stress could result in dysfunction, injury, and even disease of vital organs, particularly metabolic organs such as the liver (Bataller and Brenner, 2005; Huang C. C. et al., 2013; Huang K. C. et al., 2013; Sunny et al., 2017). Local inflammatory injury of liver tissues induced by exhaustive exercise could be a trigger of systemic inflammation (Suzuki et al., 2020). Under the EE, the liver is prone to inflammatory damage, which can further cause systemic damage. The overproduction of inflammatory cytokines can compromise the ability to resist oxidative damage, inducing cellular dysfunction and necrosis. It could eventually result in the pathologic progress of damaged tissue. Although anti-inflammatory agents have been tested for more than a decade, they still face numerous challenges (Gao and Ye, 2012; Donath, 2014; Pollack et al., 2016; Goldfine and Shoelson, 2017). For example, serious side effects caused by hypermedication and off-target effects could contribute to Cushing syndromes, gastrointestinal hemorrhage, and autoimmune disorders (Donath et al., 2013; Geer et al., 2014). Hence, few effective medications presented noteworthy effectiveness or enduring safety in experimental studies, and there is still no suitable method or approved drug to manage this situation. Therefore, novel and efficient approaches are urgently needed.

Lately, numerous studies proposed that natural polyphenols have a beneficial effect on multiple kinds of metabolic disorders (Salomone et al., 2016). It is feasible to be harvested from plants and absorbed with rare side effects. Dihydromyricetin (DHM) is one of the popular natural polyphenols which is rich in *Ampelopsis grossedentata*, especially in the leaves and stalks of vine tea (Ye et al., 2015). DHM could function as an effective anti-inflammatory and antioxidative agent (Le et al., 2016). A recent study also revealed the powerful hepatoprotective properties of DHM, which significantly ameliorated steatosis and inflammatory injury of nonalcoholic fatty liver disease (NAFLD) (Chen et al., 2015). Our previous studies indicated it could alleviate liver fibrosis *via* hepatic stellate cells inactivation through driving autophagy and immunoregulation (Zhou et al., 2019). These results indicate the potential of DHM for alleviating hepatic inflammation caused by EE. However, its poor aqueous solubility and low bioavailability limit its clinical application for liver inflammatory disease (Liu et al., 2017a; Liu et al., 2019). Therefore, there is an urgent demand to find appropriate drug carriers to improve DHM bioavailability to protect against liver injury.

Liposomes are promising pharmaceutical carriers that have attracted much attention as an efficient drug delivery system (Allen and Cullis, 2013). It is compatible with both hydrophilic and lipophilic drugs. Its chemical structure is feasible for versatile modification and adjustable characteristics to effectively improve bioavailability. Thus, it could achieve great therapeutic effects and reduce nonspecific cytotoxicity. At present, numerous liposomes for drug delivery are under research, and some are ready for clinical application (Antimisiaris et al., 2021). Long-circulating liposomes could be synthesized by grafting certain chemically and biologically inert synthetic polymers, such as PEG, to protect the liposome surface from the local environment (Allen, 1994). They have shown great advantages in dose-independent, nonsaturable, log-linear kinetics and increased bioavailability (Van Slooten et al., 2001). A recent study underlined the importance of liposomes to improve the bioavailability of DHM for killing bacteria *in vitro* (Luo et al., 2021). However, whether the system works for liver disease models *in vivo* has not yet been determined.

In this study, DHM-encapsulated PEGylated liposomes (DHM-Lipo) were prepared to elevate the bioavailability. We evaluated the feasibility of DHM-encapsulated liposomes as an effective agent for curing liver injury by employing EE-induced inflammatory liver models *in vivo*. We investigated the hepatoprotective and anti-inflammatory effects of DHM-Lipo. Using fluorescently labeled technology, we characterized the accumulated effects of DHM-Lipo on liver macrophages, which influence immune regulation and functions in the liver. As the results indicated that DHM-Lipo primarily exerts its anti-inflammatory actions by regulating macrophage polarization *in vivo*, we further used the LPS-induced inflammatory model in the RAW264.7 cell line to investigate the cell-specific mechanisms of DHM-induced macrophage polarization. These data offer insightful strategies for strengthening the regional immunomodulation and anti-inflammation of DHM on liver injury.

## 2 Materials and Methods

### 2.1 Chemicals and Reagents

DHM was purchased from Mansite Bio-Technology (China) and Sigma-Aldrich (United States). LPS and 3-TYP were purchased from MedChemExpress (United States). 1,2-distearoyl-sn-glycero-3-phosphoethanolamine-N-[biotinyl (polyethylene glycol)-2K] (PEG2K-DSPE) was obtained from Xi’an Ruixi Biological Technology (China). ELISA kits were obtained from Shanghai FANKEL Industry (China). The murine macrophage cell line RAW264.7 and exclusive complete medium (icell-m047-001b) were obtained from ICell Bioscience Inc., China. IR-808 was provided by the Institute of Combined Injury of Army Medical University.

### 2.2 Preparation of DHM-Lipo

Briefly, 93 mg soy phosphatidylcholine, 20 mg cholesterol, and 27 mg PEG2K-DSPE were dissolved in 5.6 ml dichloromethane to obtain the aqueous phase. Subsequently, 7 mg DHM was added and solubilized in 5.6 ml methanol under magnetic stirring for 30 min to produce the lipid phase. The aqueous and liquid solutions were added dropwise to 28 ml methanol/dichloromethane (4:1) mixture solvent at the same time under magnetic stirring for 5 min. The harvested solution was placed on the rotary steamer under reduced pressure rotary steamer (40°C, 80 r/min). Then, 20 ml PBS was added and processed with ultrasound. The solution was filtered by the 0.45 um film and 0.22 um film sequentially. Unencapsulated DHM was cleared by dialysis at 4°C against PBS using Slide-A-Lyzer dialysis cassettes (JingKeHongDa Biotechnology Co., China) (Bartneck et al., 2015). Ultimately, a homogeneous and translucent DHM liposome solution was obtained. The blank liposomes were also prepared by a similar method.

### 2.3 Preparation of IR-808 Labeled DHM-Lipo

To verify the distribution of DHM-Lipo *in vivo*, its surface was further labeled with a near-infrared (NIR) heptamethine cyanine dye (IR-808). IR-808 belongs to a class of Cy7 NIR probes that have been reported with strong fluorescent emission near 800 nm (Tan et al., 2012). IR-808 was synthesized according to previously established methods (Luo et al., 2016). Briefly, DHM-Lipo was mixed with IR-808 (10 mM) in 1.0 ml ultrapure water and shaken for 30 min. With hydrophobic heptamethine core and negative carboxyl terminus, IR-808 was efficiently labeled on the surface of PEG-DSPE modified liposomes through hydrophobic and electrostatic interaction. IR-808 labeled DHM-Lipo was purified by centrifuge filtration (3,000 rpm for 5 min) through centrifugal filters (10 kDa), washed three times with ultrapure water to remove free IR-808. The final product was concentrated at 1 ml and stored at 4 °C for further use.

### 2.4 Morphological Observations

The morphology of the DHM-Lipo was examined by the transmission electron microscope (TEM, TECNAI 10, Philips, United States). Briefly, the DHM-Lipo solution was added to the copper mesh with the film and stained by phosphotungstic acid negative stain solution for 2 min. TEM was used for examining the morphology of the liposomes after air drying. A laser particle size analyzer (Zetasizer Nano, MALVERN, UK) was used to analyze the particle size of DHM-Lipo.

### 2.5 Encapsulation Efficiency

The high-performance liquid chromatography (HPLC, Waters, India) was used to acquire the encapsulated efficiency by analyzing the supernatant after centrifugation of liposomes. The experiment demonstrated out on C18 column at 30°C. The encapsulation efficiency% was calculated using the following equation (Moghimipour et al., 2018).

$$\mathrm{encapsulation}\;\mathrm{efficiency}\%=100\frac{\mathrm{TD}-\mathrm{FD}}{\mathrm{TD}}$$

Whereas TD is the amount of DHM originally added to the formulation and FD is the amount of the free DHM in the supernatant after centrifugation.

### 2.6 Release Kinetics of the DHM

2 ml DHM-Lipo was supplemented into a dialysis bag (MWCO 5000 Da) and then dialyzed against 500 ml PBS at 25°C for 100 h. At designed intervals, 10 ml of sample from the reservoir was harvested, and 10 ml of fresh PBS was supplemented again. The DMH concentration of the harvested samples was analyzed by HPLC. The’ release kinetic curve was calculated to reflect the accumulated release percentage.

### 2.7 Cytocompatibility Assays

Cell viability was investigated by Cell Counting Kit-8 (CCK-8, Dojindo, Japan). Briefly, RAW264.7 cells were replated at 8000 cells/well in 96-well plates. Then, the 2 mg/ml DHM-Lipo was added. At the time point of 24 and 48 h, 10 μL of CCK-8 solution (Dojindo, Japan) were added and incubated for 2 h. The optical density (OD) value was measured at 450 nm with a microplate reader (Bio-Rad Laboratories, United States).

### 2.8 Animals and Experimental Procedures

**FIGURE 1:**
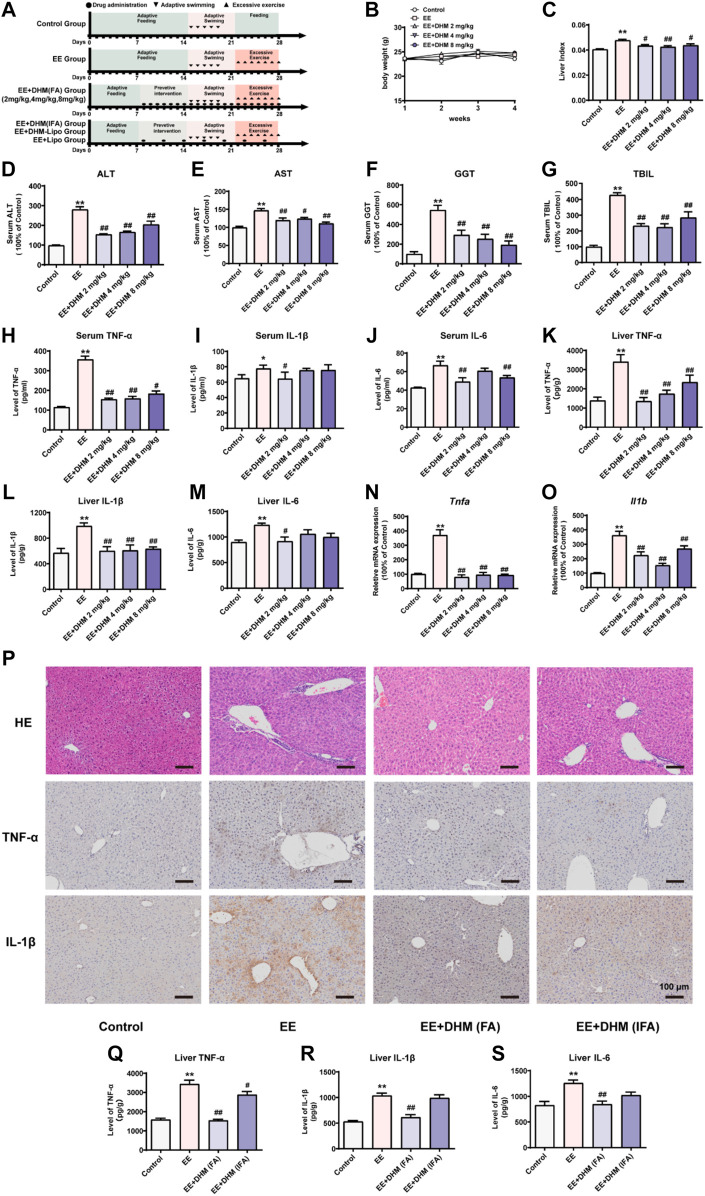
DHM administration ameliorated EE-induced liver inflammation and its efficacy was influenced by the dosing interval **(A)** Schematic diagram of the experimental design **(B)** The body weights of the mice were recorded **(C)** The liver index represents the ratio of liver weight to body weight **(D–G)** Serum levels of ALT, AST, GGT, and TBIL were examined **(H-M)** The expression of the inflammatory cytokines TNF-α, IL-1β, and IL-6 in mouse serum samples **(H–J)** and mouse liver samples **(K-M)** was examined by ELISA **(N-O)** The mRNA expression levels of *Tnfa* and *Il1b* were detected by qRT–PCR **(P)** Liver inflammation was examined by H&E and IHC for TNF-α and IL-1β **(Q-S)** The expression of the inflammatory cytokines TNF-α, IL-1β, and IL-6 in mouse liver samples was examined by ELISA. Data are presented as the mean ± SEM (*n* = 5). ^*^ *p* < 0.05, ^**^ *p* < 0.01, compared to the control group; ^#^ *p* < 0.05, ^##^ *p* < 0.01, compared to the EE group. Scale bar, 100 μm.

Male C57BL/6J mice were fed in a condition as previously reported (Zhou et al., 2021). The experimental procedure of each group was illustrated in Figure 1A. After 1 week of adaptive feeding, DHM was administered intraperitoneally for 1 week. Then, mice executed adaptive swimming exercises for 1 week and exhaustive swimming exercises for another week, following the protocol of previous studies with slight modification (Kim et al., 2014; Yuan et al., 2018). Briefly, swimming was carried out with ten mice per plastic box (90 × 50 × 40 cm) filled to a depth of 30 cm with water maintained at a temperature of 34 ± 1°C. All mice were adapted to swimming for 5 days initially: Day 1, two times of 30-s swimming with a 2 min rest interval between the swimming periods; Day 2, two times of 2 min of swimming with a 2 min rest interval; Day 3, three times of 10 min of swimming with a 5 min rest interval; Day 4, two times of 15 min of swimming with a 5 min rest interval; Day 5, a period of 30 min with no pause. After that, except for the mice in the control group, all mice started exhaustive exercise training in the fourth week. During this period, mice should swim until exhaustion once a day for seven consecutive days. Exhaustion was defined by two criteria: greater than 10 s spent below the water surface and lack of a “righting reflex” when placed on a flat surface (Dawson and Horvath, 1970; Thomas and Marshall, 1988). All efforts were made to minimize animal suffering after the experiments. Serum and liver tissues were collected and stored at 80°C. Part of the mouse was used for Flow Cytometry analysis. All the animal experiments were approved by the Animal Care and Use Committee of Third Military Medical University (Chongqing, China; Approval SYXC-2017–0002).

### 2.9 Biochemical Measurement of Serum Components

Serum was prepared by solidification and centrifugation (4 °C, 3000 g, 10min) and stored at -80 °C (Zeng et al., 2019). Biochemical measurement of alanine aminotransferase (ALT), aspartate aminotransferase (AST), γ-glutamyl transpeptidase (GGT), and total bilirubin (TBIL) was performed on an Olympus AV5400 auto analyzer.

### 2.10 Enzyme-Linked Immunosorbent Assay

TNF-α, IL-1β, and IL-6, from the cultured macrophage supernatant, the mice serum and liver samples were quantified by TNF-α (#F2132), IL-1β (#F2040) and IL-6 (#F2163) ELISA kits (Fankew, China) according to the manufacturer’s instructions. The SpectraMax^®^ M2 spectrophotometer (Molecular Devices Corp., United States) was used to obtain the OD value at 450 nm.

### 2.11 Quantitative Real-Time Polymerase Chain Reaction (qRT-PCR)

RNA was extracted with TRIzol reagent (Invitrogen, United States). We used qTower 2.2 real-time PCR system (Analytik Jena, Germany) to run qRT-PCR with SYBR Premix Ex Taq II (Takara Bio, Japan). All primers are listed in Table 1. Relative mRNA expression levels were normalized to those of β-actin and calculated by the 2^−ΔΔCt^ method.

**TABLE 1:** Sequences of primers used in qRT-PCR.

Target Gene	Primers
*Tnfa*	F: 5′-ATG​TCT​CAG​CCT​CTT​CTC​ATT​C-3′
	R: 5′-GCT​TGT​CAC​TCG​AAT​TTT​GAG​A-3′
*Il6*	F: 5′-CTC​CCA​ACA​GAC​CTG​TCT​ATA​C-3′
	R: 5′-CCA​TTG​CAC​AAC​TCT​TTT​CTC​A-3′
*Il1b*	F: 5′-AAC​TGT​GAA​ATA​GCA​GCT​TTC​G-3′
	R: 5′-CTG​TGA​GAT​TTG​AAG​CTG​GAT​G-3′
*iNos*	F: 5′-TGG​AGC​CAG​TTG​TGG​ATT​GTC-3′
	R: 5′-GGT​CGT​AAT​GTC​CAG​GAA​GTA​G-3′
*CD206*	F: 5′-CAA​GCG​ATG​TGC​CTA​CC-3′
	R: 5′-AAT​GCT​GTG​GAT​ACT​TGC​C-3′
*Sirt3*	F: 5′-CGC​TAA​ACT​TCT​CCC​GGG​TT-3′
	R: 5′-ACA​CTA​GTC​CTC​GCC​AAA​CG-3′
*Hif1α*	F: 5′-TCT​CGG​CGA​AGC​AAA​GAG​TC-3′
	R: 5′-AGC​CAT​CAT​GGG​CTT​TCA​GAT​AA-3′
*Fizz1*	F: 5′-GGG​ATG​ACT​GCT​ACT​GGG​TG-3′
	R: 5′-TCA​ACG​AGT​AAG​CAC​AGG​CA-3′
*Ym1*	F: 5′-GGG​CCC​TTA​TTG​AGA​GGA​GC-3′
	R: 5′-CCA​GCT​GGT​ACA​GCA​GAC​AA-3′
*Il10*	F: 5′-GCT​CCA​AGA​CCA​AGG​TGT​CT-3′
	R: 5′-CGG​AGA​GAG​GTA​CAA​ACG​AGG-3′
*β-actin*	F: 5′-CGA​GGC​CCC​CCT​GAA​C-3′
	R: 5′-GCC​AGA​GGC​GTA​CAG​GGA​TA-3′

### 2.12 Histological and Immunohistochemical Analyses

Immediately after animals were sacrificed, liver samples were fixed with 4% paraformaldehyde and embedded in paraffin. The hematoxylin and eosin (H&E) were performed on the liver sections.

For visualization of inflammatory cytokines distribution in the liver, the samples were then stained with TNF-α (1:20 dilution, Abcam, # Ab183218) and IL-1β (1:200 dilution, Abcam, # Ab9722). Following a standard staining protocol. The section results were scanned by a high-resolution digital slide scanner (VS-200, Olympus, Japan).

For immunofluorescence, liver frozen sections were processed using anti-F4/80 antibody (1:50 dilution, Abcam, #Ab60343). And anti-CD206 antibody (1:100 dilution, Abcam, #Ab64693), anti-iNOS antibody (1:50 dilution, Abcam, #Ab3523), anti-SIRT3 antibody (1:100 dilution, Abcam, #Ab189860), and anti-HIF-1a antibody (1:100 dilution, Abcam, #Ab228649). Then, the liver sections were treated with proteinase K (1:200, Solarbio, China) for 30 min, next by Triton X-100 (0.1%, Beyotime, China) for 30 min, and then with normal goat serum (ZSGB-BIO, China) for 30 min. Subsequently, the sections were incubated by the primary antibodies at 4 °C overnight, and then the secondary antibodies (1:1000, #Ab150165, #Ab150062, Invitrogen, United States) for 1 h at room temperature. Finally, DAPI solution (0.1%, Beyotime, China) was applied to visualize the cell nucleus for 10 min at room temperature. The immunostaining image was captured by the confocal microscopy system (LSM780, ZEISS, German) and analyzed by the matching software (Zen 2.3, ZEISS, German).

### 2.13 Cell Culture and Treatment

The cell line RAW264.7 of murine macrophage and exclusive complete medium (icell-m047-001b) were obtained from ICell Bioscience Inc., China. Cells were cultured at 37°C in a 5% CO_2_ atmosphere. To establish the model of inflammation, RAW264.7 cells were treated with LPS in sequential concentrations (0, 25, 50,100, and 200 ng/ml) for 24 h. To explore the effect of DHM, cells were preincubated with DHM at various concentrations (0, 10, 20, and 30 μM) for 2 h and thereafter treated by LPS (100 ng/ml) for 24 h. Moreover, SIRT3 inhibitor 3-TYP (50 μM) was used 1 h before DHM treatment to investigate the molecular mechanism of RAW264.7 cells in response to DHM. The small interfering RNA (siRNA) targeting Sirt3 were purchased from RiboBio (China). The sequence of the mouse siRNA was as follows: Sirt3, 5-ACU​CCC​AUU​CUU​CUU​UCA​C-3. Twenty-four hours after seeding, cells were transiently transfected with 100 nM siRNA per dish at 80% confluence using the lipofectamine 2000 (Invitrogen Life Technology, Carlsbad, CA, United States). The knockdown efficiency of the target proteins was measured with western blot assay. All experiments were repeated at least three times.

### 2.14 Flow Cytometry (FCM) Analysis

Mice were sacrificed and the liver was harvested for analysis *ex vivo* as previously described (Park et al., 2017). Cells were isolated and stained with anti-mouse CD45 (1:100 dilution, Biolegend, #103116), anti-mouse F4/80 (1:100 dilution, Biolegend, #123108), anti-mouse CD206 (1:100 dilution, Biolegend, #141720), and anti-mouse CD11c (1:100 dilution, Biolegend, #117310), and then tested using flow cytometry (LSRFortessaTM cell analyzer, BD, United States). Data were analyzed with FlowJo V10.6.

### 2.15 Western Blot Analysis

Protein expression was investigated by western blot analysis as in previous methods (Zhou et al., 2021). The primary antibodies were used to study the protein expression of sirtuins-3 (SIRT3, 1:1000 dilution; Cell Signaling Technology, #2627S), hypoxia-inducible factor-1α (HIF-1α, 1:300 dilution; Proteintech, #20960-1-AP), and β-actin (1:1000 dilution, Santa Cruz, #47778), respectively.

### 2.16 Statistical Analysis

Statistical analysis of *in vitro* and *in vivo* studies was performed using GraphPad Prism 9 (GraphPad Software Inc., CA). A one-way analysis of variance (ANOVA) was performed to determine significance by the Turkey-Kramer post hoc test, which was set at *p* < 0.05. Quantitative data are presented as the mean ± standard error of mean (X ± SEM) values. All experiments were repeated independently at least three times.

## 3 Results

### 3.1 DHM Administration Ameliorated EE-Induced Liver Inflammation and Its Efficacy Was Influenced by the Dosing Interval

Exhaustive exercise leads to liver injury caused by inflammation (Huang C. C. et al., 2013; Huang K. C. et al., 2013). Mice were randomly assigned to five groups (*n* = 15/group): control, exhaustive exercise (EE), and EE + DHM (frequent administration, FA) at 2, 4, and 8 mg/kg body weight (Figure 1A). There were no significant differences in body weight between each group (Figure 1B). The liver index represents the ratio of liver weight to body weight. The liver index of the EE group was increased compared with that of control mice, and this trend was reversed after DHM administration (*p* < 0.05, Figure 1C). Furthermore, the serum levels of ALT, AST, GGT, and TBIL in mice in the EE group were significantly elevated compared with those in mice in the control group (*p* < 0.01, Figure 1D–G, *n* = 5/group). Surprisingly, the EE-induced effect was ameliorated by DHM administration (*p* < 0.05, Figure 1D–G). Moreover, the EE-induced increase in inflammatory cytokines in the serum and liver, including tumor necrosis factor-a (TNF-α), interleukin 1β (IL-1β), and interleukin 6 (IL-6), was strongly inhibited by DHM (2 mg/kg) administration (*p* < 0.05, Figure 1H-M). Moreover, the increased hepatic mRNA levels of *Tnfa* and *Il1b* induced by EE were also significantly reversed by DHM *in vivo* (*p* < 0.01, Figure 1N–O). Overall, 2 mg/kg DHM exhibited the most hepatoprotective effect on EE-induced liver injury. Previous studies reported that poor bioavailability limits the clinical application of DHM (Liu et al., 2019). To explore the influence of bioavailability on DHM efficacy, we adjusted the dosing interval of DHM administration into two groups: EE + DHM (FA) (1 administration every day) and EE + DHM (infrequent administration, IFA) (1 administration every 3 days) (Figure 1A). As expected, EE-induced notable inflammatory infiltration was ameliorated by DHM (FA) administration, as evidenced by histology with H&E staining and immunohistochemistry (IHC) for TNF-α and IL-1β (Figure 1P). Moreover, EE-induced expression of the inflammatory cytokines TNF-α, IL-1β, and IL-6 was decreased by DHM (FA) administration, as determined by ELISA analysis (Figure 1Q–S). Regrettably, the DHM (IFA) group showed a certain difference in the expression of inflammation compare with DHM (FA) group (Figure 1P–S), suggesting that DHM (IFA) may lead to a significant compromise in the anti-inflammatory effect.

### 3.2 Physical and Cytocompatibility Characteristics of the DHM-Lipo

**FIGURE 2:**
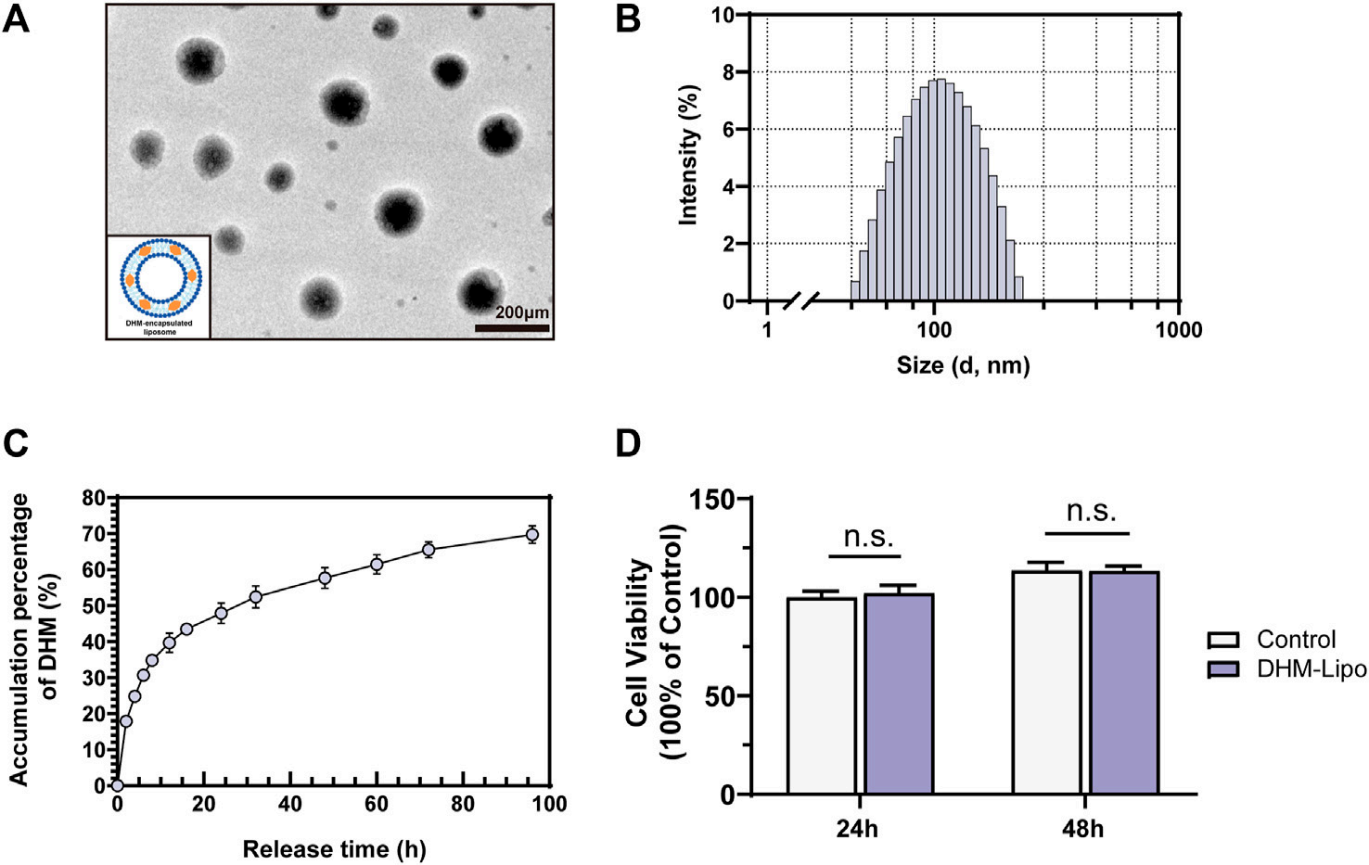
Physical and cytocompatibility characteristics of the DHM-Lipo **(A)** Micrographs of transmission electron microscopy of the DHM-Lipo **(B)** Size distributions of the DHM-Lipo **(C)** Release kinetics of DHM from the DHM-Lipo **(D)** The cytocompatibility assay of the DHM-Lipo cocultured with RAW264.7 cells was tested by CCK-8. The data are expressed as the mean ± SEM (*n* = 3). ^n. s.^ *p* > 0.05, compared to the control group. Scale bar, 200 nm.

Our results in Section 3.1 and previous studies indicate that the poor aqueous solubility and low bioavailability of DHM diminished its hepatoprotective effect (Liu et al., 2017a). Therefore, searching for an efficient drug delivery strategy to improve DHM bioavailability and prevent premature release before reaching the desired site is necessary. Liposomes have shown promising potential for drug delivery. Its outstanding performance in resisting degradation, penetrating pathogen barriers, and adjusting the release behavior makes it a valuable prospect (Liu et al., 2020). As described in the Methods section, we synthesized long-circulating liposomes for favorable DHM delivery. The micromorphologies of the DHM-Lipo were characterized by transmission electron microscopy, as depicted in Figure 2A. The liposomes exist as nanosized spheres with smooth surfaces and good dispersibility. The particle size of the tested sample presented a normal distribution, and the average particle diameter of the DHM-Lipo was 116.1 nm (Figure 2B). The release kinetics of DHM is shown in Figure 2C. Within 1 h, the DHM was released in a burst with a percentage of 17.9%. Subsequently, the sustained release percentage of DHM decreased and stabilized gradually during the middle stage of release from 2 to 24 h. At this stage, additional 30% of DHM was released from the nanoparticles. After 3 days, the cumulative release percentage reached 65.5 ± 3.1%. The encapsulation efficiency of DHM in the liposomes was 45%. This result indicates that DHM loaded in the liposomes could be released stably for an extended time. The CCK-8 assay was conducted to quantify the cytotoxicity of DHM-Lipo on RAW264.7 cells. As shown in Figure 2D, the cell proliferation activity of the DHM-Lipo group showed no obvious difference compared to that of the control group at 24 h or 48 h (*p* > 0.05). In brief, the results demonstrated that DHM-Lipo exhibited desirable characteristics of nanosized microspheres, good dispersion, stable release, and favorable cytocompatibility.

### 3.3 DHM-Lipo Administration Ameliorated EE-Induced Liver Inflammation *in vivo*

**FIGURE 3:**
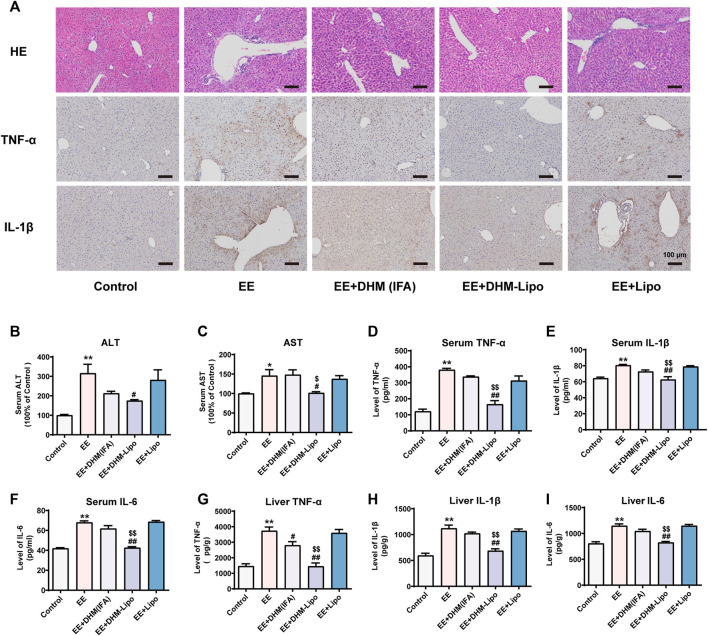
DHM-Lipo administration ameliorated EE-induced liver inflammation *in vivo* **(A)** Liver inflammation was examined by H&E and IHC for TNF-α and IL-1β **(B–C)** Serum levels of ALT and AST were examined **(D–I)** The expression of the inflammatory cytokines TNF-α, IL-1β, and IL-6 in mouse serum samples **(D–F)** and mouse liver samples **(G–I)** was examined by ELISA. Data are presented as the mean ± SEM (*n* = 5). ^*^ *p* < 0.05, ^**^ *p* < 0.01, compared to the control group; ^#^ *p* < 0.05, ^##^ *p* < 0.01, compared to the EE group; ^$^ *p* < 0.05, ^$$^ *p* < 0.01, compared to the EE + DHM (IFA) group. Scale bar, 100 μm.

As stated above, C57BL/6J mice were used to establish the EE-induced liver inflammation model. DHM (IFA), DHM-Lipo, and liposomes were administered to the EE-induced mouse model were administered every 3 days, as illustrated in Figure 1A (*n* = 5). The single intraperitoneal dose of the DHM (IFA) group was set to 2 mg/kg DHM based on Section 3.1, and the DHM-Lipo group was set at 40 mg/kg liposomal DHM. With a loading efficiency of 5% for the liposomes, the DHM content is identical. As expected, EE-induced inflammatory injury in the liver was ameliorated by infrequent administration of DHM-Lipo. Histologically, the anti-inflammatory effect of the DHM-Lipo was more powerful than that of free DHM, as evidenced by a greater reduction in the secretion of TNF-α and IL-1β. There were no significant differences between the EE group and EE + Lipo group, indicating that blank liposomes could not exert an anti-inflammatory effect on liver injury (Figure 3A). Furthermore, the serum levels of ALT and AST in mice in the EE + DHM-Lipo group were significantly decreased compared with those in the EE group (*p* < 0.05) but ALT was not decreased in the EE + DHM (IFA) group or EE + lipo group (Figure 3B,C). Moreover, EE-induced inflammatory cytokine levels in the serum (Figure 3D–F) and liver (Figure 3G–I) were significantly inhibited by DHM-Lipo administration, but not by the EE + DHM (IFA) group except for Liver TNF-α. In brief, these data demonstrate that DHM-Lipo administration notably attenuated liver injury and inflammation in EE-treated mice. The hepatoprotective effect of DHM-Lipo was more effective than that of free DHM (IFA), indicating that DHM-Lipo overcomes the limitation of poor bioavailability of DHM.

### 3.4 Biodistribution of DHM-Lipo at the Organ and Cellular Levels

**FIGURE 4:**
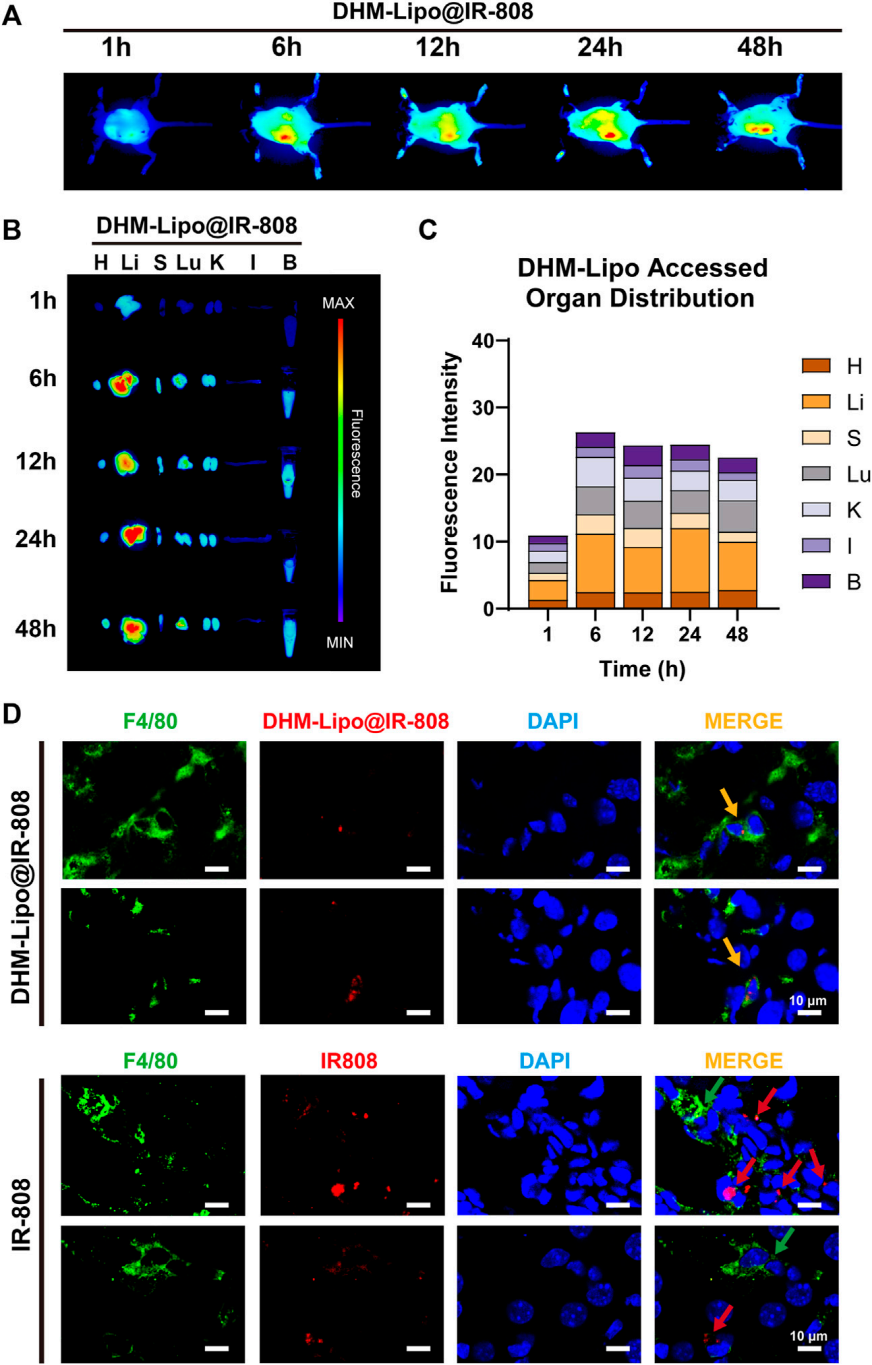
Biodistribution of DHM-Lipo at the organ and cellular levels **(A)** Preferential accumulation of DHM-Lipo@IR-808 from 1 to 48 h after intraperitoneal injection was examined by *in vivo* NIR fluorescence imaging **(B–C)** Fluorescence reflectance imaging (FRI) *ex vivo* scans of heart, liver, spleen, lung, kidney, intestine, and blood at 1, 6, 12, 24, and 48 h after intraperitoneal administration of DHM-Lipo@IR-808 at 2 mgkg^−1^. The average radiant efficiency of FRI is shown as a histogram **(D)** Colocalization of F4/80 with DHM-Lipo@IR-808 or IR-808 in liver tissue as imaged by confocal microscopy. *n* = 3, Scale bar, 10 μm.

In recent years, lipid nanoparticles have been developed to passively and actively target drugs in the liver (Böttger et al., 2020). Thus, we hypothesized that the hepatoprotective effect of the DHM-Lipo was possibly related to its liver-targeting bioactivity. To verify this hypothesis, we prepared DHM-Lipo@IR-808 and subsequently performed *in vivo* NIR fluorescence imaging to assess the liver targeting capability after intraperitoneal injection of DHM-Lipo@IR-808. The fluorescent signals associated with the liver region sites can be visualized with low background interfering fluorescence 1–48 h after injection (Figure 4A). Next, we performed fluorescence reflectance imaging (FRI) *ex vivo* scans of the heart, liver, spleen, lung, kidney, intestine, and blood. The fluorescence intensity of the dissected organs further confirmed the preferential accumulation of DHM-Lipo@IR-808 in the liver (Figure 4B). Quantification of fluorescence revealed that the liver is the predominant organ for DHM-Lipo uptake. 48 h after injection, fluorescently tagged liposomes predominantly accumulated in the liver, where they were widely distributed (Figure 4C). We also detected the distribution of DHM-Lipo@IR-808 in liver macrophages (Figure 4D). Visualization of DHM-Lipo@IR-808 *in vivo* showed that the macrophages in the liver exhibited a favorable uptake of DHM-Lipo@IR-808 at 48 h, as evidenced by DHM-Lipo@IR-808 mainly accumulating in F4/80^+^ macrophages in different samples. In contrast, no colocalization of free IR-808 and F4/80 was observed in the liver. Taken together, these results indicate that DHM-Lipo@IR-808 accumulated in hepatic macrophages.

### 3.5 DHM Treatment Promoted a Shift in Liver Macrophage Polarity

**FIGURE 5:**
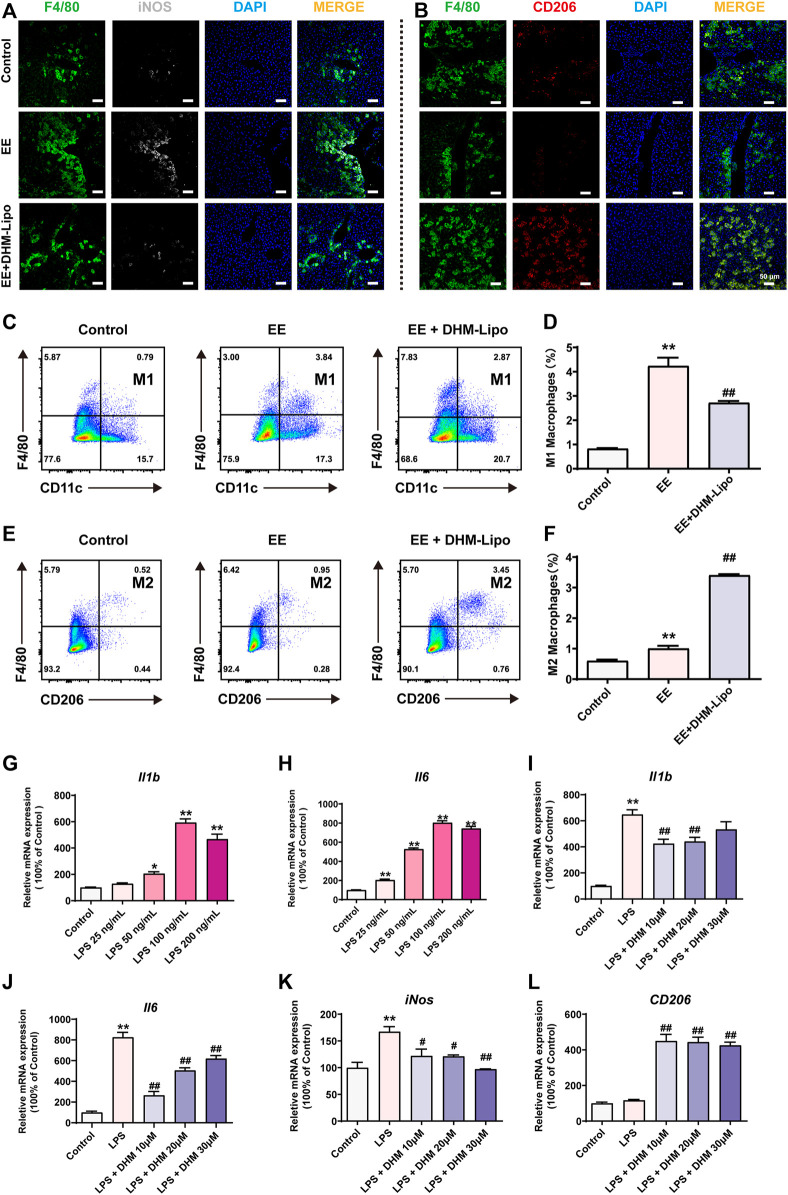
DHM treatment promoted a shift in liver macrophage polarity **(A)** Representative immunofluorescence images of F4/80 (green, a macrophage marker) and iNOS (white, M1 type) **(B)** Representative immunofluorescence images of F4/80 (green) and CD206 (red, M2 type) **(C–F)** The frequency of M1-type macrophages (cells gated by CD45^+^F4/80^+^CD11c^+^) and M2-type macrophages (cells gated by CD45^+^F4/80^+^CD206^+^) by FCM **(G–H)** RAW264.7 cells were treated with a series of concentrations (0, 25, 50, 100, and 200 ng/ml) of LPS, and the mRNA expression of *Il1b* and *Il6* was detected by qRT–PCR **(I-L)** Preincubation with a series of concentrations (0, 10, 20, and 30 μM) of DHM for 2 h before treatment with LPS (100 ng/ml) for 24 h. The mRNA expression levels of *Il1b*, *Il6*, *iNOS,* and *CD206* were detected by qRT–PCR. Data are presented as the mean ± SEM (*n* = 3). ^*^ *p* < 0.05, ^**^ *p* < 0.01, compared to the control group; ^#^ *p* < 0.05, ^##^ *p* < 0.01, compared to the LPS group. Scale bar = 50 µm.

To further investigate the effects of DHM-Lipo on liver macrophages, we performed immunofluorescence staining for M1 and M2 macrophage markers in liver tissue. Staining of hepatic M1 macrophages with the specific marker of colocalization of iNOS with F4/80 revealed that DHM-Lipo reduced the amount of M1 macrophages in the liver (Figure 5A). DHM-Lipo treatment was almost exclusively related to reduced numbers of M1 but not M2 macrophages, as evidenced by an increase in the colocalization of CD206 with F4/80 that was induced by DHM-Lipo (Figure 5B). This phenomenon was confirmed by flow cytometric determination (Figure 4C–F). We analysed M1 (CD45^+^ F4/80^+^ CD11c^+^) and M2 (CD45^+^ F4/80^+^ CD206^+^) macrophages from the liver tissues of mice by flow cytometry according to previous study (Zhang X. et al., 2019). DHM-Lipo significantly decreased the percentage of M1-like macrophages within the total macrophage population that was activated by EE (*p* < 0.01, Figure 5C,D). Simultaneously, DHM-Lipo increased the percentage of M2-like macrophages (*p* < 0.01, Figure 5E,F). These results demonstrate that M1 macrophages (inflammatory) in the liver are polarized towards the alternatively activated (anti-inflammatory) M2 subtype.

To further verify the effect of DHM on macrophage polarization *in vitro*, we established an inflammatory cell model using RAW264.7 cells treated with a series of concentrations (0, 25, 50, 100, and 200 ng/ml) of lipopolysaccharides (LPS) for 24 h. Compared to the control group, the mRNA expression of *Il1b* and *Il6* was significantly increased, especially in the 100 ng/ml group (Figure 5G,H). To further explore the anti-inflammatory effect of DHM, RAW264.7 cells were preincubated with a series of concentrations (0, 10, 20, and 30 μM) of DHM for 2 h before treatment with LPS (100 ng/ml) for 24 h. Compared with treatment with LPS alone, DHM administration, especially at 10 μM, significantly suppressed the LPS-induced increase in *Il1b* and *Il6* mRNA expression (*p* < 0.01, Figure 5I,J). In addition, the mRNA expression of *iNos*, which represents M1-like macrophages, was significantly increased in the LPS group and was significantly reversed by DHM, as expected (*p* < 0.05, Figure 5K). Moreover, the mRNA expression of *CD206, Fizz1*, *Ym1,* and *Il10,* which represent M2-like macrophages, was significantly increased in the LPS + DHM groups compared with the LPS group (*p* < 0.05, Figure 5L, Supplementary Figure S1). Collectively, these results demonstrate that DHM administration can regulate macrophage polarization. DHM effectively inhibited M1-like macrophages and enhanced M2-like macrophages *in vivo* and *in vitro*.

### 3.6 DHM Regulated Macrophage Polarity Through the SIRT3/HIF-1α Signaling Pathway

**FIGURE 6:**
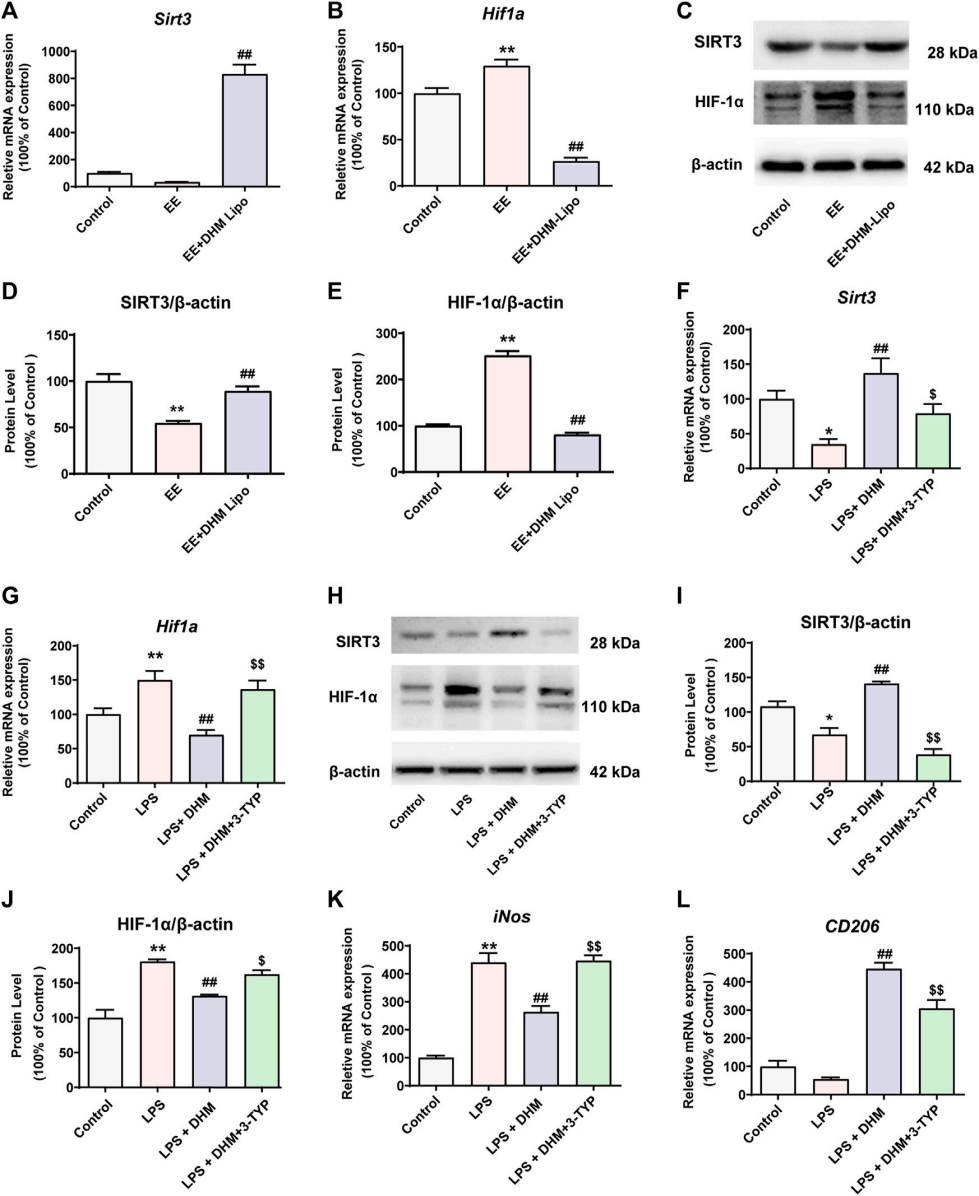
DHM regulated macrophage polarity through the SIRT3/HIF-1α signaling pathway **(A-E)** The mRNA and protein expression levels of SIRT3 and HIF-1α in liver tissue were detected by qRT–PCR(A-B) and western blot(C). Bar charts showing the quantification of SIRT3/β-actin (D) and HIF-1α/β-actin (E) **(F-G)** RAW264.7 cells were treated with DHM (10 μM) for 2 h, and then the cells were exposed to LPS (100 ng/ml) for an additional 24 h. The SIRT3 inhibitor 3-TYP (50 μM) was added 1 h before DHM treatment. The mRNA expression levels of *Sirt3* (F) and *Hif1a* (G) were detected by qRT–PCR **(H-J)** The expression levels of SIRT3 and HIF-1α were detected by western blot(H). Bar charts showing the quantification of SIRT3/β-actin (I) and HIF-1α/β-actin (J) **(K-L)** The mRNA expression levels of *iNos* (K) and *CD206* (L) were detected by qRT–PCR. Data are presented as the mean ± SEM (*n* = 3). ^*^ *p* < 0.05, ^**^ *p* < 0.01, compared to the control group; ^##^ *p* < 0.01, compared to the LPS group; ^$^ *p* < 0.05, ^$$^ *p* < 0.01, compared to the LPS + DHM group.

Many studies have found that the polarization phenotype of immune cells is related to their energy metabolism (Orihuela et al., 2016). SIRT3 is a classical regulatory molecule of energy metabolism that is involved in the regulatory mechanism of a variety of immune cell phenotypes in previous studies (Nogueiras et al., 2012). Furthermore, HIF-1α, as an important target of SIRT3, has also been shown to be involved in the regulation of the immune cell phenotype (Nogueiras et al., 2012). Thus, we investigated whether SIRT3 and HIF-1α are involved in the regulatory effect of DHM on macrophage polarization. *In vivo,* the mRNA and protein expression levels of SIRT3 were notably increased in the EE + DHM-Lipo group compared with the EE group (*p* < 0.01, Figure 6A,C,D). However, the mRNA and protein expression of HIF-1α was increased in the EE group and decreased in the EE + DHM-Lipo group (*p* < 0.01, Figure 6B,C,E). As expected, comparable results were shown in the mRNA and protein expression levels of SIRT3 and HIF-1α *in vitro*. However, these benefits of DHM were abolished by treatment with 3-TYP, a SIRT3 inhibitor (Figure 5F–J), and Sirt3 siRNA (Supplementary Figure S2). Moreover, the DHM-induced elimination of the expression of the M1-like macrophage marker (*iNos*) and the upregulation of the expression of the M2-like macrophage marker (*CD206, Fizz1, Ym1,* and *Il10*) in LPS-treated RAW264.7 cells was abolished by treatment with 3-TYP (Figure 6K, L, Supplementary Figure S3). Collectively, these results demonstrate that DHM suppresses M1-like macrophages and upregulates M2-like macrophages by activating the SIRT3/HIF-1α signaling pathway.

## 4 Discussion

**FIGURE 7:**
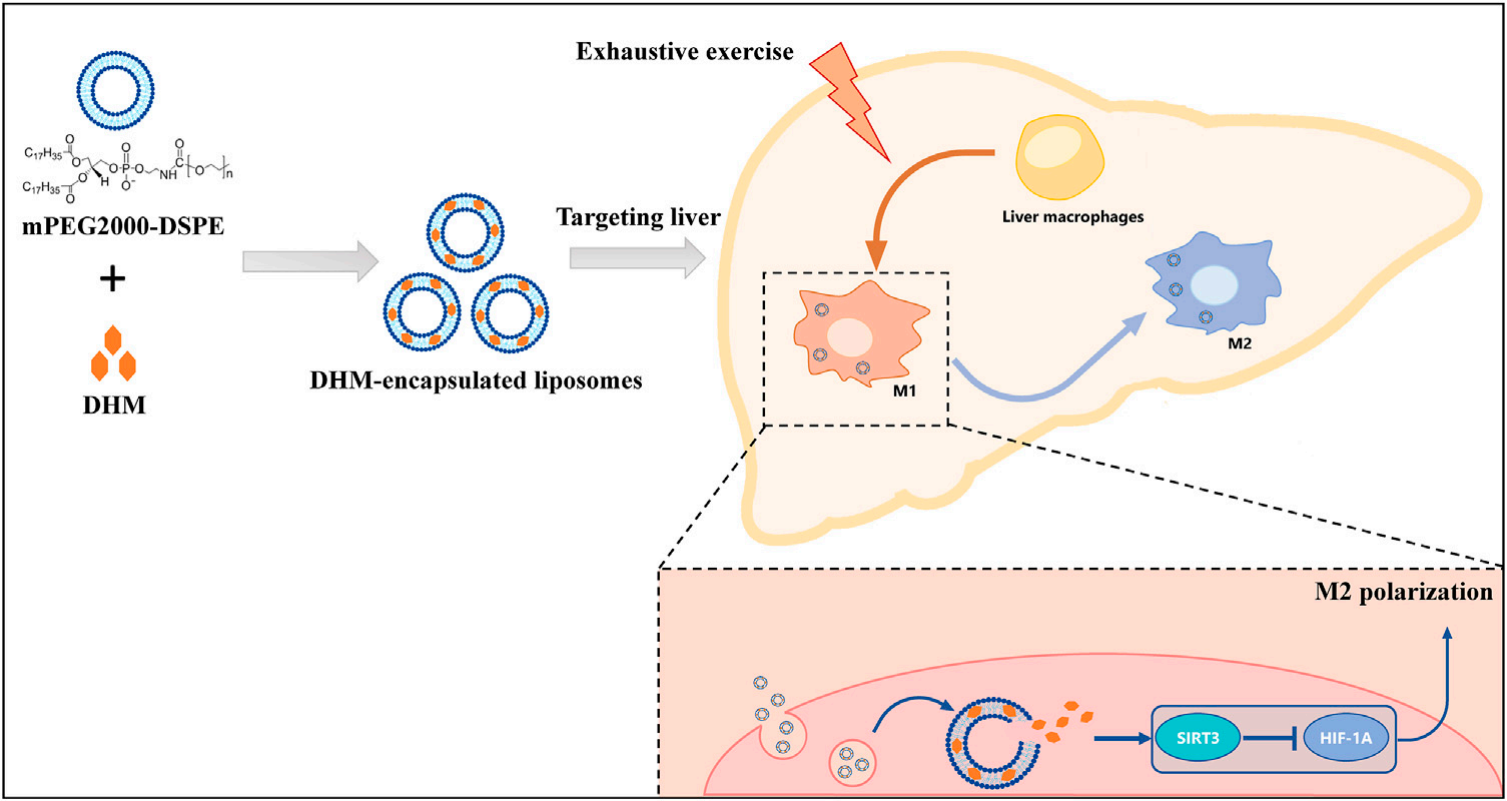
Schematic diagram. The preventive and therapeutic effect of DHM-Lipo against EE-induced liver injury occurs through the regulation of macrophage polarity from the M1 to M2 type *via* the SIRT3/HIF-1α pathway.

At present, there is still a lack of suitable anti-inflammatory agents for the treatment of liver injury and the resulting dysfunction caused by exercise stress. In this study, we evaluated the anti-inflammatory efficacy and mechanism of liposomal DHM on inflammatory injury induced by EE for the first time. The primary finding of this study was that DHM-encapsulated liposomes administration significantly reduced liver inflammation through long-term targeting of the liver and sustained DHM release. Such effects elevate the bioavailability of DHM and overcome its dominating clinical limitation. In addition, liposomal DHM promoted macrophage polarization to an anti-inflammatory phenotype by activating the SIRT3/HIF-1α signaling pathway (Figure 7). Our study demonstrated that liposomal DHM could be a novel preventive and therapeutic strategy for the future treatment of exercise-induced liver injury.

Exercise load-induced injuries are characterized by inflammatory changes caused by unbalanced metabolic homeostasis. As the main metabolic organ of the human body, the liver is prone to an inflammatory response to injury. Effective control and improvement of liver inflammation is the main prevention and treatment strategy for sports injuries. The important role of nutrition intervention in the prevention and treatment of exercise injuries has attracted the attention of researchers (Williams et al., 2019; Yada et al., 2019). Dietary nutrition intervention plays a key role in preventing and treating liver inflammatory diseases (Del Ben et al., 2017). Our previous study of a dietary intervention found that DHM can effectively improve blood lipids and blood sugar, reduce insulin resistance and oxidative stress, and inhibit inflammatory damage in NAFLD patients (Zeng et al., 2019), which is consistent with our results (Figure 1). The major disadvantages associated with DHM use are chemical instability and poor bioavailability. DHM is slightly soluble in water at room temperature (0.2 mg/ml at 25°C), which is the main cause of its poor membrane permeability (Peff = (1.84 ± 0.37) × 10^–6^ cm/s) and bioavailability (Wang et al., 2016; Liu et al., 2017a). Our data also revealed that extending the dosing interval markedly affected the hepatoprotective efficacy of DHM (Figure 1P–S). This is a determinant factor that limits the pharmacological effects and clinical application of DHM. Special attention has been given to the metabolic pathways of DHM, and different approaches have been carried out to increase DHM bioavailability in both the aqueous and lipid phases (Liu et al., 2019). We have prepared a new drug delivery system for DHM using long-circulating liposomes. Ideal sustained drug delivery is one critical principle for an effective drug carrier. Our result demonstrated that DHM-encapsulated liposomes have a quite lower burst release within 2 h. Then, the liposomes released the DHM stably from 6 to 24 h. The rate of cumulative release in the 3 days was 69.7%. Overall, the DHM-Lipo here exhibited desired long-term drug release and met the demand for elevating DHM bioavailability (Figure 2). As expected, liposomal DHM showed desirable bioavailability and effective anti-inflammatory bioactivity *in vivo* even with an extended dosing interval. This was evidenced by the ability of the DHM-Lipo to ameliorate the liver injury and reduce the inflammatory cytokine deposition that was induced by EE (Figure 3). Therefore, liposomal DHM is an effective drug delivery system as an anti-inflammatory agent against EE-induced liver injury.

Interestingly, regional immunity could play a key role in EE-induced inflammation. Moreira et al. found that a single bout of prolonged, intense exercise transiently modifies a large number of immune variables (Chen et al., 2015). Our research has also found that high-intensity running-induced gastrointestinal symptoms are closely associated with a reduced percentage of ILC3 and IL-22 levels in lamina propria lymphocytes (Hou et al., 2020). Thus, the regulation of regional immunity is vital for resisting EE-induced liver injury to achieve anti-inflammation and maintain metabolic homeostasis. The mechanism of dietary nutrition intervention on liver inflammation could be due to their role in regional immunity. For example, we recently reported that the beneficial biological effects of DHM are inseparable from its immune regulatory function (Zhou et al., 2021), which is consistent with this study. Surprisingly, the DHM-Lipo accumulated in hepatic macrophages with long-term efficacy. A previous study demonstrated that DHM might be metabolized and eliminated in the intestinal tract (Liu et al., 2017a). Another study reported that DHM was distributed rapidly in various tissues, especially in the gastrointestinal tract, and was able to cross the blood−brain barrier. The elimination of DHM was almost completed within 12 h (Fan et al., 2017). Liposomal encapsulation changed the basic pharmacokinetic characteristics of DHM, which caused DHM to be distributed and metabolized mainly in the liver (Figure 4). Particularly, liver macrophages cells are thought to be the main cellular component responsible for nanoparticle accumulation in the liver (Samuelsson et al., 2017). They are located at the sinusoidal endothelium, which was preferentially targeted by lipid nanoparticles (>100 nm) (Böttger et al., 2020). Meanwhile, they have been widely recognized for their intrinsic role in particle endocytosis *via* scavenger receptors (Moghimi and Hunter, 2001). This was the pharmacokinetic basis of the anti-inflammatory mechanism of DHM, namely, regulating liver macrophage polarization.

Liver macrophages are abundant immune cells in human and murine liver tissue (Bian et al., 2020; Luo et al., 2018). The function of macrophages in altering liver tissue inflammation has attracted increasing attention. Liver macrophages are divided into two phenotypes, which have substantial functional heterogeneity (Dou et al., 2019). One of the phenotypes is classically activated (M1-like) macrophages, which produce proinflammatory mediators, such as tumor necrosis factor α (TNF-α), interleukin-6 (IL-6), and interleukin-1β (IL-1β) (Li et al., 2018). The other is alternatively activated (M2-like) macrophages, which secrete anti-inflammatory cytokines, such as IL-10, and typically express the mannose receptors CD206 and CD163 (Xi et al., 2021). Multiple studies have found that regulating the polarization phenotype of macrophages affects the outcome of diseases. For example, research has found that failure of alternative M2 activation leads to classical macrophage activation, elevated weight gain, and obesity with concurrent adipose inflammation and insulin resistance (Jung et al., 2018). Our data showed that DHM plays an important role in preventing liver inflammation by regulating M1/M2 hepatic macrophage polarization *in vivo* and *in vitro*. In particular, DHM pretreatment activated resident/infiltrating hepatic macrophages into M2 hepatic macrophages and deterred the M1 hepatic macrophage polarization induced by EE or LPS treatment (Figure 5). It is certainly reasonable because a similar function could be found in other flavonols such as quercetin and myricetin, which have a similar molecular structure with DHM (Lu et al., 2018; Yao et al., 2020). Thus, the shift in liver macrophage polarity enhanced M2-like macrophages and inhibited M1-like macrophages, which is a critical therapeutic target for EE-induced liver inflammation.

To further explore the mechanism of DHM-regulated hepatic macrophage polarization, we found different energy metabolism pathways between the two phenotypes of macrophages. Under normal circumstances, hepatic macrophages are in a stable state, and the metabolic phenotype is dominated by oxidative phosphorylation. When hepatic macrophages polarize to M1, the metabolic phenotype changes to glycolysis, and the tricarboxylic acid cycle is inhibited (O'Neill, 2015). M2 hepatic macrophages mainly obtain energy through oxidative phosphorylation, which promotes pyruvate entry into mitochondria for oxidative phosphorylation and activation of the electron transport chain (Mills and O'Neill, 2016). In recent years, studies have found that the phenotype of a variety of immune cells is affected by their energy metabolism (Olenchock et al., 2017). SIRT3 is a member of the sirtuin family of NAD^+^-dependent deacetylases located in the mitochondria and is an important molecular switch of energy metabolism (Morigi et al., 2018). SIRT3 plays an important role in various chronic diseases, such as obesity, cardiovascular disease, NASH, and NAFLD (Kane and Sinclair, 2018). Studies have reported that SIRT3 can regulate hypoxia-inducible factor-1 (HIF-1α), which is the key molecule of energy metabolism during the immune phenotypic polarization of macrophages. A previous study revealed SIRT3 inhibits the glycolysis metabolic pathway by inhibiting HIF-1α (Zhang H. X. et al., 2019) and researchers also found that reduction in HIF-1α binding to the IL-1β promoter and the subsequent downregulation of IL-1β expression inhibited the polarization of macrophages to M1 and promoted M2 (Palsson-McDermott et al., 2015). Combined with our previous studies, DHM can activate SIRT3 in a variety of models (Liu et al., 2017b; Liu et al., 2018). Therefore, we speculate that DHM may regulate the immunophenotype of hepatic macrophages by activating the SIRT3/HIF-1α signaling pathway. We confirmed that under LPS treatment, DHM upregulated the expression of SIRT3 and inhibited HIF-1α. However, after 3-TYP and Sirt3 siRNA treatment, the effect of DHM was significantly inhibited (Figure 6 and Supplementary Figure S2). The macrophages tended to increase the expression of M1 markers and decrease the expression of M2 markers (Figure 6 and Supplementary Figure S3). Collectively, these results demonstrate that DHM-induced hepatic macrophage polarization is mediated partially through the activation of the SIRT3/HIF-1α signaling pathways.

There are still several limitations in our research. Firstly, the majority of our *in vivo* data was based on mice with liver inflammation caused by the exhaustive exercise of swimming. Whether DHM-encapsulated liposomes protected the liver from other pathological injuries, such as mice fed a high-fat diet, needs to be explored further. Besides, the present study mainly focused on the anti-inflammatory effect of DHM-encapsulated liposomes. The physico-chemical characteristics and other biological functions of DHM-encapsulated liposomes are worth exploring in the future. At last, whether other immune regulation mechanisms contributed to the positive effect of DHM on regulating macrophage polarization, such as other immune cells that may have crosstalk with macrophages, should be investigated.

## 5 Conclusion

In this study, we synthesized a long-circling DHM-encapsulated liposome with good bioavailability that could target the liver in the long term and sustain DHM delivery. The primary finding of this study was that DHM-encapsulated liposomes significantly reduced the liver inflammation induced by EE by promoting macrophage polarization from the M1 to M2 subtype by activating the SIRT3/HIF-1α signaling pathway (Figure 7). These results provide important evidence uncovering the potential immunoregulatory efficiency of DHM for preventing and treating liver inflammation. Additionally, our study demonstrated that liposomal DHM could be a novel preventive and therapeutic strategy for the future treatment of exercise-induced liver injury.

## Data Availability

The raw data supporting the conclusions of this article will be made available by the authors, without undue reservation.

## Abbreviations

3-TYP, 3-(1H-1,2,3-triazol-4-yl) pyridine; ALT, alanine transaminase; AST, aspartate transaminase; BSA, bovine serum albumin; CCK-8, Cell Counting Kit-8; DHM, dihydromyricetin; EE, exhaustive exercise; ELISA, enzyme-linked immunosorbent assay; FCM, flow cytometry; FITC, fluorescein isothiocyanate; GGT, γ-glutamyl transpeptidase; HIF-1α, hypoxia-inducible factor-1α; H&E, hematoxylin and eosin; LDH, lactate dehydrogenase; LPS, lipopolysaccharides; mRNA, messenger ribonucleic acid; NAFLD, non-alcoholic fatty liver disease; NASH, nonalcoholic steatohepatitis; qRT-PCR, quantitative real-time polymerase chain reaction; SIRT3, silent mating type information regulation2 homolog-3; siRNA, small interfering RNA; TBIL, total bilirubin.

## References

[B1] Allen T. M. Cullis P. R. (2013). Liposomal Drug Delivery Systems: from Concept to Clinical Applications. Adv. Drug Deliv. Rev. 65 (1), 36–48. 10.1016/j.addr.2012.09.037 23036225

[B2] Allen T. M. (1994). Long-circulating (Sterically Stabilized) Liposomes for Targeted Drug Delivery. Trends Pharmacol. Sci. 15 (7), 215–220. 10.1016/0165-6147(94)90314-x 7940982

[B3] Antimisiaris S. G. Marazioti A. Kannavou M. Natsaridis E. Gkartziou F. Kogkos G. (2021). Overcoming Barriers by Local Drug Delivery with Liposomes. Adv. Drug Deliv. Rev. 174, 53–86. 10.1016/j.addr.2021.01.019 33539852

[B4] Bartneck M. Scheyda K. M. Warzecha K. T. Rizzo L. Y. Hittatiya K. Luedde T. (2015). Fluorescent Cell-Traceable Dexamethasone-Loaded Liposomes for the Treatment of Inflammatory Liver Diseases. Biomaterials 37, 367–382. 10.1016/j.biomaterials.2014.10.030 25453965

[B5] Bataller R. Brenner D. A. (2005). Liver Fibrosis. J. Clin. Invest. 115 (2), 209–218. 10.1172/JCI24282 15690074 PMC546435

[B6] Bian Z. Gong Y. Huang T. Lee C. Z. W. Bian L. Bai Z. (2020). Deciphering Human Macrophage Development at Single-Cell Resolution. Nature 582 (7813), 571–576. 10.1038/s41586-020-2316-7 32499656

[B7] Böttger R. Pauli G. Chao P. H. Al Fayez N. Hohenwarter L. Li S. D. (2020). Lipid-Based Nanoparticle Technologies for Liver Targeting. Adv. Drug Deliv. Rev. 154-155, 79–101. 10.1016/j.addr.2020.06.017 32574575

[B8] Chen S. Zhao X. Wan J. Ran L. Qin Y. Wang X. (2015). Dihydromyricetin Improves Glucose and Lipid Metabolism and Exerts Anti-Inflammatory Effects in Nonalcoholic Fatty Liver Disease: A Randomized Controlled Trial. Pharmacol. Res. 99, 74–81. 10.1016/j.phrs.2015.05.009 26032587

[B9] Dawson C. A. Horvath S. M. (1970). Swimming in Small Laboratory Animals. Med. Sci. Sports 2 (2), 51–78. 10.1249/00005768-197000220-00002 4939286

[B10] Del Ben M. Polimeni L. Baratta F. Pastori D. Angelico F. (2017). The Role of Nutraceuticals for the Treatment of Non-Alcoholic Fatty Liver Disease. Br. J. Clin. Pharmacol. 83 (1), 88–95. 10.1111/bcp.12899 26852185 PMC5338137

[B11] Donath M. Y. Dalmas É. Sauter N. S. Böni-Schnetzler M. (2013). Inflammation in Obesity and Diabetes: Islet Dysfunction and Therapeutic Opportunity. Cell Metab. 17 (6), 860–872. 10.1016/j.cmet.2013.05.001 23747245

[B12] Donath M. Y. (2014). Targeting Inflammation in the Treatment of Type 2 Diabetes: Time to Start. Nat. Rev. Drug Discov. 13 (6), 465–476. 10.1038/nrd4275 24854413

[B13] Dou L. Shi X. He X. Gao Y. (2019). Macrophage Phenotype and Function in Liver Disorder. Front. Immunol. 10, 3112. 10.3389/fimmu.2019.03112 32047496 PMC6997484

[B14] Fan L. Tong Q. Dong W. Yang G. Hou X. Xiong W. (2017). Tissue Distribution, Excretion, and Metabolic Profile of Dihydromyricetin, a Flavonoid from Vine Tea (Ampelopsis Grossedentata) after Oral Administration in Rats. J. Agric. Food Chem. 65 (23), 4597–4604. 10.1021/acs.jafc.7b01155 28534405

[B65] Fan W. Evans R. M. (2017). Exercise Mimetics: Impact on Health and Performance. Cell Metab. 25 (2), 242–247. 27889389 10.1016/j.cmet.2016.10.022 PMC5555683

[B15] Gao Z. G. Ye J. P. (2012). Why Do Anti-Inflammatory Therapies Fail to Improve Insulin Sensitivity? Acta Pharmacol. Sin. 33 (2), 182–188. 10.1038/aps.2011.131 22036866 PMC3270211

[B16] Geer E. B. Islam J. Buettner C. (2014). Mechanisms of Glucocorticoid-Induced Insulin Resistance: Focus on Adipose Tissue Function and Lipid Metabolism. Endocrinol. Metab. Clin. North Am. 43 (1), 75–102. 10.1016/j.ecl.2013.10.005 24582093 PMC3942672

[B17] Goldfine A. B. Shoelson S. E. (2017). Therapeutic Approaches Targeting Inflammation for Diabetes and Associated Cardiovascular Risk. J. Clin. Invest. 127 (1), 83–93. 10.1172/JCI88884 28045401 PMC5199685

[B18] Hou P. Zhou X. Yu L. Yao Y. Zhang Y. Huang Y. (2020). Exhaustive Exercise Induces Gastrointestinal Syndrome through Reduced ILC3 and IL-22 in Mouse Model. Med. Sci. Sports Exerc 52 (8), 1710–1718. 10.1249/MSS.0000000000002298 32079925

[B19] Huang C. C. Huang W. C. Yang S. C. Chan C. C. Lin W. T. (2013). Ganoderma Tsugae Hepatoprotection against Exhaustive Exercise-Induced Liver Injury in Rats. Molecules 18 (2), 1741–1754. 10.3390/molecules18021741 23434860 PMC6270576

[B20] Huang K. C. Wu W. T. Yang F. L. Chiu Y. H. Peng T. C. Hsu B. G. (2013). Effects of Freshwater Clam Extract Supplementation on Time to Exhaustion, Muscle Damage, Pro/anti-Inflammatory Cytokines, and Liver Injury in Rats after Exhaustive Exercise. Molecules 18 (4), 3825–3838. 10.3390/molecules18043825 23531600 PMC6270442

[B21] Jung S. B. Choi M. J. Ryu D. Yi H. S. Lee S. E. Chang J. Y. (2018). Reduced Oxidative Capacity in Macrophages Results in Systemic Insulin Resistance. Nat. Commun. 9 (1), 1551. 10.1038/s41467-018-03998-z 29674655 PMC5908799

[B22] Kane A. E. Sinclair D. A. (2018). Sirtuins and NAD(+) in the Development and Treatment of Metabolic and Cardiovascular Diseases. Circ. Res. 123 (7), 868–885. 10.1161/CIRCRESAHA.118.312498 30355082 PMC6206880

[B23] Kim S. H. Kim E. K. Choi E. J. (2014). High-Intensity Swimming Exercise Increases Dust Mite Extract and 1-Chloro-2,4-Dinitrobenzene-Derived Atopic Dermatitis in BALB/c Mice. Inflammation 37 (4), 1179–1185. 10.1007/s10753-014-9843-z 24526290

[B24] Le L. Jiang B. Wan W. Zhai W. Xu L. Hu K. (2016). Metabolomics Reveals the Protective of Dihydromyricetin on Glucose Homeostasis by Enhancing Insulin Sensitivity. Sci. Rep. 6, 36184. 10.1038/srep36184 27796348 PMC5087077

[B25] Li X. Jin Q. Yao Q. Xu B. Li L. Zhang S. (2018). The Flavonoid Quercetin Ameliorates Liver Inflammation and Fibrosis by Regulating Hepatic Macrophages Activation and Polarization in Mice. Front. Pharmacol. 9, 72. 10.3389/fphar.2018.00072 29497376 PMC5819566

[B26] Liu Y. Gao D. Zhang X. Liu Z. Dai K. Ji B. (2016). Antitumor Drug Effect of Betulinic Acid Mediated by Polyethylene Glycol Modified Liposomes. Mater Sci. Eng. C Mater Biol. Appl. 64, 124–132. 10.1016/j.msec.2016.03.080 27127036

[B27] Liu L. Yin X. Wang X. Li X. (2017). Determination of Dihydromyricetin in Rat Plasma by LC-MS/MS and its Application to a Pharmacokinetic Study. Pharm. Biol. 55 (1), 657–662. 10.1080/13880209.2016.1266669 27951743 PMC6130699

[B28] Liu L. Wan J. Lang H. Si M. Zhu J. Zhou Y. (2017). Dihydromyricetin Delays the Onset of Hyperglycemia and Ameliorates Insulin Resistance without Excessive Weight Gain in Zucker Diabetic Fatty Rats. Mol. Cell Endocrinol. 439, 105–115. 10.1016/j.mce.2016.10.028 27984083

[B29] Liu L. Zhou M. Lang H. Zhou Y. Mi M. (2018). Dihydromyricetin Enhances Glucose Uptake by Inhibition of MEK/ERK Pathway and Consequent Down-Regulation of Phosphorylation of PPARγ in 3T3-L1 Cells. J. Cell Mol. Med. 22 (2), 1247–1256. 10.1111/jcmm.13403 29160030 PMC5783835

[B30] Liu D. Mao Y. Ding L. Zeng X. A. (2019). Dihydromyricetin: A Review on Identification and Quantification Methods, Biological Activities, Chemical Stability, Metabolism and Approaches to Enhance its Bioavailability. Trends Food Sci. Technol. 91, 586–597. 10.1016/j.tifs.2019.07.038 32288229 PMC7127391

[B31] Liu W. Hou Y. Jin Y. Wang Y. Han J. (2020). Research Progress on Liposomes: Application in Food, Digestion Behavior and Absorption Mechanism. Trends Food Sci. Technol. 104, 177–189. 10.1016/j.tifs.2020.08.012

[B32] Lu H. Wu L. Liu L. Ruan Q. Zhang X. Hong W. (2018). Quercetin Ameliorates Kidney Injury and Fibrosis by Modulating M1/M2 Macrophage Polarization. Biochem. Pharmacol. 154, 203–212. 10.1016/j.bcp.2018.05.007 29753749

[B33] Luo S. Yang Z. Tan X. Wang Y. Zeng Y. Wang Y. (2016). Multifunctional Photosensitizer Grafted on Polyethylene Glycol and Polyethylenimine Dual-Functionalized Nanographene Oxide for Cancer-Targeted Near-Infrared Imaging and Synergistic Phototherapy. ACS Appl. Mater Interfaces 8 (27), 17176–17186. 10.1021/acsami.6b05383 27320692

[B34] Luo X. Li H. Ma L. Zhou J. Guo X. Woo S. L. (2018). Expression of STING Is Increased in Liver Tissues from Patients with NAFLD and Promotes Macrophage-Mediated Hepatic Inflammation and Fibrosis in Mice. Gastroenterology 155 (6), 1971–1984. 10.1053/j.gastro.2018.09.010 30213555 PMC6279491

[B35] Luo F. Zeng D. Chen R. Zafar A. Weng L. Wang W. (2021). PEGylated Dihydromyricetin-Loaded Nanoliposomes Coated with Tea Saponin Inhibit Bacterial Oxidative Respiration and Energy Metabolism. Food Funct. 12 (19), 9007–9017. 10.1039/d1fo01943k 34382988

[B36] Mills E. L. O'Neill L. A. (2016). Reprogramming Mitochondrial Metabolism in Macrophages as an Anti-Inflammatory Signal. Eur. J. Immunol. 46 (1), 13–21. 10.1002/eji.201445427 26643360

[B37] Moghimi S. M. Hunter A. C. (2001). Recognition by Macrophages and Liver Cells of Opsonized Phospholipid Vesicles and Phospholipid Headgroups. Pharm. Res. 18 (1), 1–8. 10.1023/a:1011054123304 11336343

[B38] Moghimipour E. Rezaei M. Ramezani Z. Kouchak M. Amini M. Angali K. A. (2018). Folic Acid-Modified Liposomal Drug Delivery Strategy for Tumor Targeting of 5-Fluorouracil. Eur. J. Pharm. Sci. 114, 166–174. 10.1016/j.ejps.2017.12.011 29247686

[B39] Morigi M. Perico L. Benigni A. (2018). Sirtuins in Renal Health and Disease. J. Am. Soc. Nephrol. 29 (7), 1799–1809. 10.1681/ASN.2017111218 29712732 PMC6050939

[B40] Nogueiras R. Habegger K. M. Chaudhary N. Finan B. Banks A. S. Dietrich M. O. (2012). Sirtuin 1 and Sirtuin 3: Physiological Modulators of Metabolism. Physiol. Rev. 92 (3), 1479–1514. 10.1152/physrev.00022.2011 22811431 PMC3746174

[B41] Olenchock B. A. Rathmell J. C. Vander Heiden M. G. (2017). Biochemical Underpinnings of Immune Cell Metabolic Phenotypes. Immunity 46 (5), 703–713. 10.1016/j.immuni.2017.04.013 28514672 PMC5660630

[B42] O'Neill L. A. (2015). A Broken Krebs Cycle in Macrophages. Immunity 42 (3), 393–394. 10.1016/j.immuni.2015.02.017 25786167

[B43] Orihuela R. McPherson C. A. Harry G. J. (2016). Microglial M1/M2 Polarization and Metabolic States. Br. J. Pharmacol. 173 (4), 649–665. 10.1111/bph.13139 25800044 PMC4742299

[B44] Palsson-McDermott E. M. Curtis A. M. Goel G. Lauterbach M. A. Sheedy F. J. Gleeson L. E. (2015). Pyruvate Kinase M2 Regulates Hif-1α Activity and IL-1β Induction and Is a Critical Determinant of the Warburg Effect in LPS-Activated Macrophages. Cell Metab. 21 (1), 65–80. 10.1016/j.cmet.2014.12.005 25565206 PMC5198835

[B45] Park J. K. Shao M. Kim M. Y. Baik S. K. Cho M. Y. Utsumi T. (2017). An Endoplasmic Reticulum Protein, Nogo-B, Facilitates Alcoholic Liver Disease through Regulation of Kupffer Cell Polarization. Hepatology 65 (5), 1720–1734. 10.1002/hep.29051 28090670 PMC5397326

[B46] Pollack R. M. Donath M. Y. LeRoith D. Leibowitz G. (2016). Anti-Inflammatory Agents in the Treatment of Diabetes and its Vascular Complications. Diabetes Care 39 (Suppl. 2), S244–S252. 10.2337/dcS15-3015 27440839

[B47] Salomone F. Godos J. Zelber-Sagi S. (2016). Natural Antioxidants for Non-Alcoholic Fatty Liver Disease: Molecular Targets and Clinical Perspectives. Liver Int. 36 (1), 5–20. 10.1111/liv.12975 26436447

[B48] Samuelsson E. Shen H. Blanco E. Ferrari M. Wolfram J. (2017). Contribution of Kupffer Cells to Liposome Accumulation in the Liver. Colloids Surf. B Biointerfaces 158, 356–362. 10.1016/j.colsurfb.2017.07.014 28719856 PMC5645238

[B49] Sunny N. E. Bril F. Cusi K. (2017). Mitochondrial Adaptation in Nonalcoholic Fatty Liver Disease: Novel Mechanisms and Treatment Strategies. Trends Endocrinol. Metab. 28 (4), 250–260. 10.1016/j.tem.2016.11.006 27986466

[B50] Suzuki K. Tominaga T. Ruhee R. T. Ma S. (2020). Characterization and Modulation of Systemic Inflammatory Response to Exhaustive Exercise in Relation to Oxidative Stress. Antioxidants (Basel) 9 (5), 401. 10.3390/antiox9050401 PMC7278761 32397304

[B51] Tan X. Luo S. Wang D. Su Y. Cheng T. Shi C. (2012). A NIR Heptamethine Dye with Intrinsic Cancer Targeting, Imaging and Photosensitizing Properties. Biomaterials 33 (7), 2230–2239. 10.1016/j.biomaterials.2011.11.081 22182749

[B52] Thomas D. P. Marshall K. I. (1988). Effects of Repeated Exhaustive Exercise on Myocardial Subcellular Membrane Structures. Int. J. Sports Med. 9 (4), 257–260. 10.1055/s-2007-1025017 3182155

[B53] Van Slooten M. L. Boerman O. Romøren K. Kedar E. Crommelin D. J. Storm G. (2001). Liposomes as Sustained Release System for Human Interferon-Gamma: Biopharmaceutical Aspects. Biochim. Biophys. Acta 1530 (2-3), 134–145. 10.1016/s1388-1981(00)00174-8 11239816

[B54] Wang C. Tong Q. Hou X. Hu S. Fang J. Sun C. C. (2016). Enhancing Bioavailability of Dihydromyricetin through Inhibiting Precipitation of Soluble Cocrystals by a Crystallization Inhibitor. Cryst. Growth Des. 16 (9), 5030–5039. 10.1021/acs.cgd.6b00591

[B55] Williams N. C. Killer S. C. Svendsen I. S. Jones A. W. (2019). Immune Nutrition and Exercise: Narrative Review and Practical Recommendations. Eur. J. Sport Sci. 19 (1), 49–61. 10.1080/17461391.2018.1490458 29975589

[B56] Xi S. Zheng X. Li X. Jiang Y. Wu Y. Gong J. (2021). Activated Hepatic Stellate Cells Induce Infiltration and Formation of CD163(+) Macrophages via CCL2/CCR2 Pathway. Front. Med. (Lausanne) 8, 627927. 10.3389/fmed.2021.627927 33614685 PMC7893116

[B57] Yada K. Roberts L. A. Oginome N. Suzuki K. (2019). Effect of Acacia Polyphenol Supplementation on Exercise-Induced Oxidative Stress in Mice Liver and Skeletal Muscle. Antioxidants (Basel) 9 (1), 29. 10.3390/antiox9010029 PMC7022702 31905679

[B58] Yao Q. Li S. Li X. Wang F. Tu C. (2020). Myricetin Modulates Macrophage Polarization and Mitigates Liver Inflammation and Fibrosis in a Murine Model of Nonalcoholic Steatohepatitis. Front. Med. (Lausanne) 7, 71. 10.3389/fmed.2020.00071 32195263 PMC7065264

[B59] Ye L. Wang H. Duncan S. E. Eigel W. N. O'Keefe S. F. (2015). Antioxidant Activities of Vine Tea (Ampelopsis Grossedentata) Extract and its Major Component Dihydromyricetin in Soybean Oil and Cooked Ground Beef. Food Chem. 172, 416–422. 10.1016/j.foodchem.2014.09.090 25442572

[B60] Yuan X. Xu S. Huang H. Liang J. Wu Y. Li C. (2018). Influence of Excessive Exercise on Immunity, Metabolism, and Gut Microbial Diversity in an Overtraining Mice Model. Scand. J. Med. Sci. Sports 28 (5), 1541–1551. 10.1111/sms.13060 29364545

[B61] Zeng X. Yang J. Hu O. Huang J. Ran L. Chen M. (2019). Dihydromyricetin Ameliorates Nonalcoholic Fatty Liver Disease by Improving Mitochondrial Respiratory Capacity and Redox Homeostasis through Modulation of SIRT3 Signaling. Antioxid. Redox Signal 30 (2), 163–183. 10.1089/ars.2017.7172 29310441

[B62] Zhang X. Fan L. Wu J. Xu H. Leung W. Y. Fu K. (2019). Macrophage P38α Promotes Nutritional Steatohepatitis through M1 Polarization. J. Hepatology 71 (1), 163–174. 10.1016/j.jhep.2019.03.014 30914267

[B63] Zhang H. X. Li Y. N. Wang X. L. Ye C. L. Zhu X. Y. Li H. P. (2019). Probucol Ameliorates EMT and Lung Fibrosis through Restoration of SIRT3 Expression. Pulm. Pharmacol. Ther. 57, 101803. 10.1016/j.pupt.2019.101803 31085231

[B66] Zhou X. Yang J. Zhou M. Zhang Y. Liu Y. Hou P. (2019). Resveratrol Attenuates Endothelial Oxidative Injury by Inducing Autophagy *via* the Activation of Transcription Factor EB. Nutr. Metab. (Lond) 16, 42. 10.1186/s12986-019-0371-6 31303889 PMC6604179

[B64] Zhou X. Yu L. Zhou M. Hou P. Yi L. Mi M. (2021). Dihydromyricetin Ameliorates Liver Fibrosis via Inhibition of Hepatic Stellate Cells by Inducing Autophagy and Natural Killer Cell-Mediated Killing Effect. Nutr. Metab. (Lond) 18 (1), 64. 10.1186/s12986-021-00589-6 34147124 PMC8214786

