# Erratum: Dihydromyricetin-encapsulated liposomes inhibit exhaustive exercise-induced liver inflammation by orchestrating M1/M2 macrophage polarization

**DOI:** 10.3389/fphar.2022.1003574

**Published:** 2022-08-31

**Affiliations:** Frontiers Media SA, Lausanne, Switzerland

**Keywords:** dihydromyricetin, exhaustive exercise, liposome, liver inflammation, macrophage polarization

Due to a production error, there was a mistake in Figure 1 as published. An incorrect version of Figure 1P was used in the original article. The corrected Figure 1 appears below.

**FIGURE 1:**
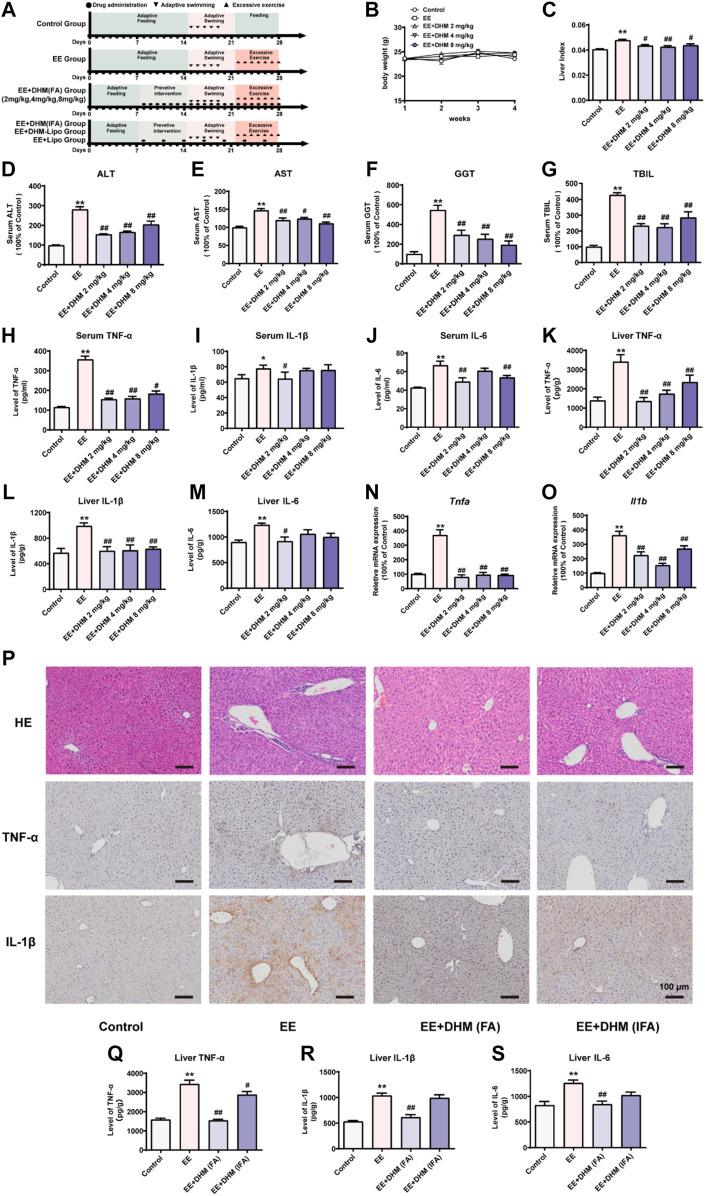
DHM administration ameliorated EE-induced liver inflammation and its efficacy was influenced by the dosing interval **(A)** Schematic diagram of the experimental design **(B)** The body weights of the mice were recorded **(C)** The liver index represents the ratio of liver weight to body weight **(D–G)** Serum levels of ALT, AST, GGT, and TBIL were examined **(H–M)** The expression of the inflammatory cytokines TNF-α, IL-1β, and IL-6 in mouse serum samples **(H–J)** and mouse liver samples **(K–M)** was examined by ELISA **(N–O)** The mRNA expression levels of Tnfa and Il1b were detected by qRT–PCR **(P)** Liver inflammation was examined by H&E and IHC for TNF-α and IL-1β **(Q–S)** The expression of the inflammatory cytokines TNF-α, IL-1β, and IL-6 in mouse liver samples was examined by ELISA. Data are presented as the mean ± SEM (*n* = 5). *p < 0.05, **p < 0.01, compared to the control group; ^#^ *p* < 0.05, ^##^ *p* < 0.01, compared to the EE group. Scale bar, 100 μm.

The publisher apologizes for this mistake. The original version of this article has been updated.

