# Supplementary figures and images for: Dihydromyricetin-Encapsulated Liposomes Inhibit Exhaustive Exercise-Induced Liver Inflammation by Orchestrating M1/M2 Macrophage Polarization
Source: Front Pharmacol. 2022 Jun 2;13:887263. doi: 10.3389/fphar.2022.887263 (PMC9205249; doi:10.3389/fphar.2022.887263)

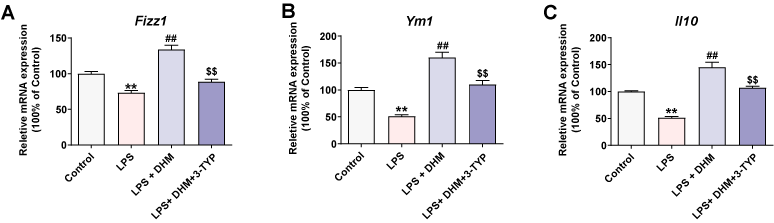

Supplement: Supplementary file 1 [file Image3.TIF]

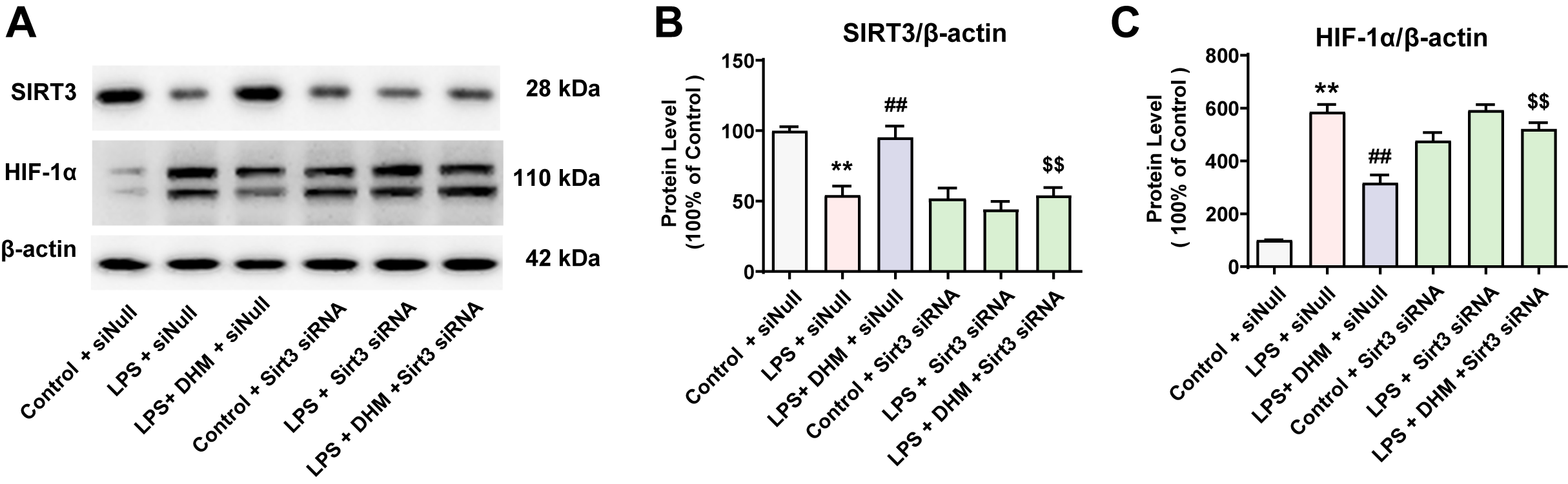

Supplement: Supplementary file 2 [file Image2.TIF]

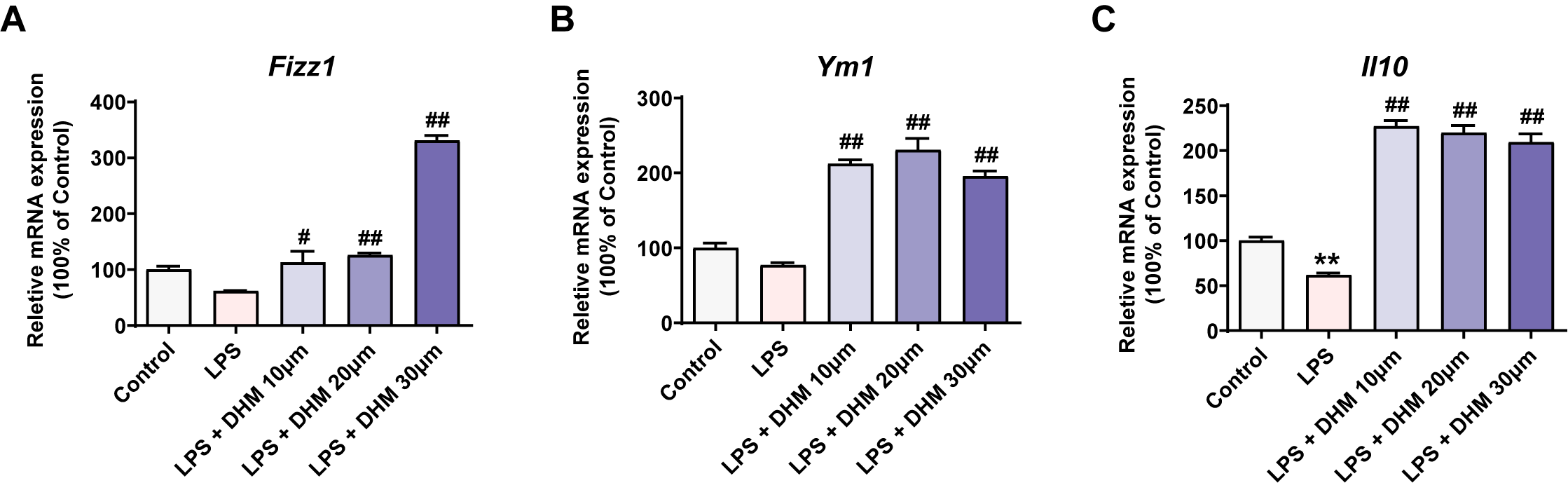

Supplement: Supplementary file 3 [file Image1.TIF]
